# Targeting Inhibition of Accumulation and Function of Myeloid-Derived Suppressor Cells by Artemisinin via PI3K/AKT, mTOR, and MAPK Pathways Enhances Anti-PD-L1 Immunotherapy in Melanoma and Liver Tumors

**DOI:** 10.1155/2022/2253436

**Published:** 2022-06-22

**Authors:** Mengqi Zhang, Lulu Wang, Wan Liu, Tian Wang, Francesco De Sanctis, Lifang Zhu, Guizhong Zhang, Jian Cheng, Qin Cao, Jingying Zhou, Aldo Tagliabue, Vincenzo Bronte, Dehong Yan, Xianchun Wan, Guang Yu

**Affiliations:** ^1^School of Basic Medical Science, Jinzhou Medical University, Jinzhou 121000, China; ^2^Guangdong Immune Cell Therapy Engineering and Technology Research Center, Center for Protein and Cell-Based Drugs, Institute of Biomedicine and Biotechnology, Shenzhen Institutes of Advanced Technology, Chinese Academy of Sciences, Shenzhen 518055, China; ^3^Department of Hematology and Oncology, Shenzhen Children's Hospital, Shenzhen 518036, China; ^4^Department of Medicine, Immunology Section, University of Verona, Verona, Italy; ^5^School of Life Science and Technology, Jinan University, Guangzhou 510632, China; ^6^University of Chinese Academy of Sciences, Beijing 100864, China; ^7^School of Biomedical Sciences, The Chinese University of Hong Kong, Hong Kong; ^8^Shenzhen BinDeBioTech Co., Ltd., Shenzhen 518055, China

## Abstract

Despite the remarkable success and efficacy of immune checkpoint blockade (ICB) therapy such as anti-PD-L1 antibody in treating cancers, myeloid-derived suppressor cells (MDSCs) that lead to the formation of the protumor immunosuppressive microenvironment are one of the major contributors to ICB resistance. Therefore, inhibition of MDSC accumulation and function is critical for further enhancing the therapeutic efficacy of anti-PD-L1 antibody in a majority of cancer patients. Artemisinin (ART), the most effective antimalarial drug with tumoricidal and immunoregulatory activities, is a potential option for cancer treatment. Although ART is reported to reduce MDSC levels in 4T1 breast tumor model and improve the therapeutic efficacy of anti-PD-L1 antibody in T cell lymphoma-bearing mice, how ART influences MDSC accumulation, function, and molecular pathways as well as MDSC-mediated anti-PD-L1 resistance in melanoma or liver tumors remains unknown. Here, we reported that ART blocks the accumulation and function of MDSCs by polarizing M2-like tumor-promoting phenotype towards M1-like antitumor one. This switch is regulated via PI3K/AKT, mTOR, and MAPK signaling pathways. Targeting MDSCs by ART could significantly reduce tumor growth in various mouse models. More importantly, the ART therapy remarkably enhanced the efficacy of anti-PD-L1 immunotherapy in tumor-bearing mice through promoting antitumor T cell infiltration and proliferation. These findings indicate that ART controls the functional polarization of MDSCs and targeting MDSCs by ART provides a novel therapeutic strategy to enhance anti-PD-L1 cancer immunotherapy.

## 1. Introduction

Immune checkpoint blockade (ICB) therapy has revolutionized the field of tumor immunotherapy [1]. Programmed cell death-1 (PD-1) expression is most highly upregulated on exhausted T cells, and engagement of PD-1 with its ligand PD-L1 leads to suppression of T cell immune responses [2–4]. High expression of PD-L1 on both antigen-presenting cells (APCs) and tumor cells not only triggers the PD-1/PD-L1 axis of T cells but also activates PD-L1/CD80 interactions on dendritic cells (DCs), leading to immunosuppression [5]. In recent years, anti-PD-L1 immunotherapy has been shown to robustly activate the immune system, produce antitumor immune responses, and improve survival outcomes in patients with various cancers including melanoma and hepatocellular carcinoma [6–8]. However, only a minority of patients experience dramatic tumor regression in response to ICB therapy, and most patients initially respond to but later become resistant to these therapies [9]. The main reason of this resistance is the presence of protumor immunosuppressive microenvironment, which inhibits effector T cells and decreases T cell infiltration into the tumor tissue [10–12].

Myeloid-derived suppressor cells (MDSCs) contribute to the formation of the tumor immunosuppressive microenvironment. High frequencies of MDSCs within tumors prior to or after ICB therapy are generally associated with unfavorable clinical outcomes, and thus, MDSC levels could predict the responsiveness or resistance to ICB therapy in cancer patients [1, 13, 14]. MDSCs are a group of heterogeneous cells consisting of precursors of DCs, macrophages, and granulocytes in the tumor microenvironment [15–17]. A large number of MDSCs are amplified in the blood, spleen, and tumor tissues of tumor-bearing mice or cancer patients [18, 19]. Murine MDSCs are defined as cells coexpressing Gr-1 and CD11b [20]. According to differential Gr-1 antigen expression of Ly6G and Ly6C epitopes, murine MDSCs can be divided into granulocyte-like MDSCs (G-MDSCs, CD11b^+^Ly6G^+^Ly6C^low/int^) and monocyte-like MDSCs (M-MDSCs, CD11b^+^Ly6G^−^Ly6C^high^) [21–23]. MDSCs mainly inhibit T cell function by producing arginase I (*Arg1*), reactive oxygen species (ROS), and immunosuppressive cytokines such as IL-10 and TGF*β* through activating STAT3, C/EBP*β*, PI3K, and MAPK signaling pathways [24–29], which skews MDSCs into M2-like tumor-promoting phenotype [30, 31]. We and others recently found that modifying gene expression patterns of MDSCs is conducive to converting an M2-like immunosuppressive phenotype to M1-like a stimulatory one, while inhibition of MDSC accumulation and functions is critical for a success anti-PD-L1 immunotherapy. Nevertheless, the current preclinical studies have shown that the drugs reducing MDSC aggregation could only partially improve the efficacy of anti-PD-L1 treatment, and the main reason is that these drugs have the single target, easy to produce drug resistance and side effects [32–34].

Artemisinins (ARTs) are a class of sesquiterpene lactones containing peroxy groups and have become the most effective antimalaria drugs without side effects [35, 36]. Recent reports showed that ARTs also have antitumor and immunomodulatory effects, which indicates the potential option for cancer treatment [37]. ART could reduce MDSC levels and enhance the antitumor immune response in 4T1 breast tumor model *in vitro* and *in vivo* [38]. In addition, our group also found that ART blocks the MDSC immunosuppression and improves the efficacy of anti-PD-L1 antibody in T cell lymphoma-bearing mice [39, 40]. However, how ART influences MDSC accumulation, function, and molecular pathways in melanoma or liver tumors and the subsequent effects on PD-L1 blockade-mediated tumor immunotherapy remains unknown. In this study, we therefore investigate the effects of ART on MDSCs *in vitro* and in B16F10 and Hepa1-6 *in vivo* tumor models and determine whether and how targeting MDSCs by ART may enhance anti-PD-L1 immunotherapy.

## 2. Materials and Methods

### 2.1. Reagent and Antibodies

DMEM, RPMI-1640, fetal bovine serum (FBS), penicillin-streptomycin (PS), Trypsin-EDTA, and phosphate-buffered saline (PBS) were purchased from Gibco. Murine GM-CSF and IL-6 from PeproTech, artemisinin (HY-B0094) from MedChemExpress, InVivoMab anti-mouse PD-L1 antibody (clone 10F.9G2) from BioXCell, dimethyl sulfoxide (DMSO, D2650) and concanavalin A (Con A, C2272) from Sigma-Aldrich, and carboxyfluorescein succinimidyl ester (CFSE) from Invitrogen were obtained. The following are fluorescein-conjugated anti-mouse antibodies: Gr-1-PerCP-Cy5.5 (clone RB6-8C5), Gr-1-FITC (clone RB6-8C5), CD11b-APC (clone M1/70), CD11b-PE (clone M1/70), CD45-Brilliant Violet 510 (clone 30-F11), Ly6C-PE-Cy7 (clone HK1.4), Ly6G-APC-Cy7 (clone 1A8), CD11c-Brilliant Violet 421 (clone N418), F4/80-FITC (clone BM8), CD3-APC (clone 17A2), CD3-FITC (clone 17A2), CD4-APC-Cy7 (clone GK1.5), CD4-APC (clone GK1.5), CD8a-PE (clone 53-6.7), CD8a-Brilliant Violet 605 (clone 53-6.7), CD19-Alexa Fluor 700 (clone 6D5), NK1.1-PE-Cy7 (clone PK136), CD25-APC (clone 3C7), DR5-PE (clone MD5-1), Annexin V-FITC, and propidium iodide (PI) solution were from Biolegend. LIVE/DEAD™ Fixable Violet Dead Cell Stain Kit was from Invitrogen, and Foxp3-PE (clone R16-715) was from BD Pharmingen.

### 2.2. Cell Culture

B16F10 and Hepa1-6 cells were obtained from cell bank, Chinese Academy of Sciences. It was subcultured using DMEM with 10% FBS and 1% PS and grown in a 37°C, 5% CO_2_ incubator. The medium was changed every 2-3 days, and cell passage was done once the cell grew to about 90% confluence.

### 2.3. Generation of Bone Marrow- (BM-) Derived MDSCs In Vitro

BM cells were isolated from wild-type C57BL/6 mice, and then, the red blood cells of isolated cells were lysed using ammonium-chloride-potassium (ACK, Beyotime) buffer. 2 × 10^6^ bone marrow cells were cultured in RPMI-1640 medium containing 10% FBS and 1% PS with murine GM-CSF (40 ng/ml) and IL-6 (40 ng/ml) for 3 days and then were treated with different concentrations of ART (100 *μ*M, 300 *μ*M, and 500 *μ*M) for another 12 hours, and the solvent DMSO was used as the control. These MDSCs were referred to as in vitro ART-treated MDSCs and DMSO-treated MDSCs, respectively.

### 2.4. Flow Cytometrical Analysis

For MDSC purification, *in vitro* cultured bone marrow cells were incubated with anti-mouse Gr-1 biotin antibody (Biolegend), but single-cell suspensions from tumors of Hepa 1-6-bearing mice were incubated with anti-mouse CD45 biotin antibody (Biolegend) followed by anti-biotin beads (Biolegend) on a MojoSort Magnet (Biolegend). These cells then were stained with cell surface marker CD11b and Gr-1 monoclonal antibodies for purification of CD11b^+^Gr-1^+^ cells on BD FACS Aria III cell sorter (BD Biosciences).

For MDSC apoptosis assay, *in vitro* cultured BM-derived ART-treated MDSCs and DMSO-treated MDSCs were stained with CD11b and Gr-1 monoclonal antibodies, Annexin V and PI molecular probes, or DR5 monoclonal antibodies. Then, cells gated on CD11b^+^Gr-1^+^ were analyzed for the percentages of Annexin V^+^ MDSCs or DR5 mean fluorescence intensity (MFI) on Beckman Coulter CytoFLEX flow cytometry and analyzed with FlowJo software (version 10, TreeStar).

For flow cytometry detection of leukocytes in tumor tissues, MDSCs were CD45^+^CD11c^−^F4/80^−^CD11b^+^Gr-1^+^ cells; M-MDSCs were CD45^+^CD11c^−^F4/80^−^CD11b^+^ Ly6G^−^Ly6C^high^ cells; G-MDSCs were CD45^+^CD11c^−^F4/80^−^CD11b^+^Ly6G^+^Ly6C^low/int^ cells; DCs were CD45^+^F4/80^−^CD11c^+^ cells; macrophages were CD45^+^CD11c^−^ F4/80^+^ cells; CD3^+^ T cells were CD45^+^CD3^+^ cells; CD4^+^ T cells were CD45^+^CD3^+^CD4^+^CD8^−^ cells; CD8^+^ T cells were CD45^+^CD3^+^CD4^−^CD8^+^ cells; B cells were CD45^+^CD19^+^ cells; and NK cells were CD45^+^CD3^−^NK1.1^+^ cells. These leukocytes were analyzed on Sony flow cytometry and analyzed with FlowJo software (version 10, TreeStar).

### 2.5. T Cell Proliferation Experiments

For *in vitro* mouse MDSC function experiments, *in vitro* BM-derived MDSCs or *in vivo* tumor MDSCs isolated from Hepa 1-6-bearing mice were cocultured at different ratios (1 : 1, 1 : 2, 1 : 4, and 1 : 8) with CFSE-labeled spleen CD3^+^ T cells purified from wild-type C57BL/6 mice activated with Con A (5 *μ*g/ml). The cells were cultured for 3 days and stained with CD3 antibody, and T cell proliferation was analyzed by flow cytometry. Data was expressed as the percentages of T cell proliferation as tested by CFSE fluorescence compared to activated T cells in the absence of MDSCs.

### 2.6. RNA Sequencing (RNA-seq) and Gene Set Enrichment Analysis (GSEA)

CD45^+^CD11b^+^Gr-1^+^ MDSCs from tumors of B16F10-bearing C57BL/6 mice treated with DMSO or ART were enriched with CD45 magnet microbeads and sorted on BD FACS Aria III cell sorter (BD Biosciences). The purity of MDSCs was >95%. RNA-seq was performed on BGISEQ500 platform (BGI-Shenzhen), RNA-seq data were aligned using bowtie2 against mm10 version of the mouse genome, and RSEM v1.2.12 software was used to estimate the raw read counts using Ensemble v84 gene information. DESeq2 was used to estimate the significance of differential expression between sample groups. Differentially expressed genes were identified as those that satisfy both Student's *t* test nominal *P* value of <0.05 and have a mean log_2_ FoldChange of ≥1. For the analysis of M2 signatures, GSEA was performed using the Broad Institute's GSEA program. M2 signature was referred from M2 gene set reported [41].

### 2.7. RT-qPCR Gene Expression Analyses

Tumor CD45^+^CD11b^+^ Gr-1^+^ MDSCs of B16F10-bearing mice were sorted on BD FACS Aria III cell sorter (BD Biosciences), and the cells were pelleted for RNA isolation using RNAiso plus (Takara). The extracted RNA was converted to cDNA with PrimeScript™ RT Master Mix (Takara). The transcript level of different genes of interest was evaluated via CFX96™ Real-Time System C1000 Touch Thermal cycler (Bio-Rad) using TB Green® Premix Ex Taq™ II (Takara). RT-qPCR reaction conditions were as follows: (1) 95°C, 5 min; (2) 95°C, 15 s; (3) 60°C, 45 s; (4) 65°C, 5 s; and (5) 95°C, 50 s; 40 cycles. Relative expression was calculated using the *Δ*∆Ct method and normalized to the reference gene *β*-actin. The primer sequences are listed in Supplementary Table S1.

### 2.8. Protein Extraction and Immunoblotting

Tumor CD45^+^CD11b^+^Gr-1^+^ MDSCs of B16F10-bearing mice that received various treatments were lysed on ice using RIPA lysis buffer supplemented with 1× complete protease inhibitors mixture and 1× phosphatase inhibitor (Roche). Cell lysates were centrifuged at 12,000 *g*, 4°C for 20 min, and the supernatant was collected to determine the protein concentration using the BCA protein Assay kit (Beyotime). 1x sample SDS buffer was added to the supernatant for electrophoresis. 30-50 *μ*g of protein per lane alongside a prestained molecular weight protein marker (GenStar) was separated on an 8% gel prepared from SDS-PAGE kit (Beyotime) and electrotransferred to immunoblot PVDF membrane (Millipore) for protein blotting. After blocking of the membrane in a western quick block kit (Beyotime) for at least 1 h, it was incubated in primary antibodies against p-RIPK3 (T231+S232) from Abcam; RIPK3, ERK, and AKT from Proteintech; p-STAT1 (Tyr701) and ARG1 from BD Biosciences; Caspase-3, Cleaved Caspase-3, iNOS, p-AKT (Ser473), p-p70 S6K (Thr389), p-ERK (Thr202/Tyr204), and p-p38 MAPK (Thr180/Tyr182) from Cell Signaling Technology; STAT1, p47^phox^, p38 MAPK, p70 S6K, and *β*-actin from Santa Cruz; and GAPDH (Bioworld Technology) with gentle agitation overnight at 4°C. HRP-conjugated secondary antibodies were used to incubate the membrane for an hour followed by protein detection with Immobilon Crescendo Western HRP substrate (Merck) and viewed on Amersham Imager 600 (GE Healthcare).

### 2.9. Cytokine Detection

ELISA kits (Biolegend, DAKEWE) were used to determine the concentrations of TGF-*β*, IL-6, IL-10, and TNF-*α* in the lysates or culture supernatants of MDSCs.

### 2.10. Arginase Enzymatic Activity and NO Production

Tumor CD45^+^CD11b^+^ Gr-1^+^ MDSCs of B16F10-bearing mice that received various treatments were lysed on ice using 100 *μ*l RIPA lysis buffer supplemented with 1× complete protease inhibitors mixture and 1× phosphatase inhibitor (Roche) for 20 minutes. The lysates were centrifuged at 4°C, 12000 rpm/min for 10 minutes. The protein lysates were diluted into ddH_2_O at 40 : 1. Then, 40 *μ*l diluents were added to each tube. 8 *μ*l of 500 mM L-arginine and 2 *μ*l of 10 mM MnCl_2_ were added to the sample group. 50 *μ*l ddH_2_O as blank control group and 50 *μ*l of 1 mM urea standard as positive control group were prepared. All the tubes were incubated in 37°C biochemical incubator for 2 hours. The reaction was stopped with 900 *μ*l (H_2_SO_4_ (98%): H_3_PO_3_ (85%): ddH_2_O = 1 : 3 : 7, vol:vol:vol). In the negative control group (sample blank), 8 *μ*l L-arginine and 2 *μ*l MnCl_2_ were added without 37°C incubation. Lastly, 10 *μ*l colour solution (9% ISPF) was added to each tube and incubated in a dry bath at 95°C for 30 minutes after oscillating and mixing. The urea concentration was measured by the absorbance of each tube in 562 nm. One unit of enzyme activity is defined as the amount of enzyme that catalyzed the formation of 1 *μ*mol urea per min.

To detect NO production, DAF-FM DA (Beyotime) was diluted at 1 : 1000 using DAF-FM DA diluent buffer provided by the kit to make the final concentration of 5 *μ*mol/L, and then, 0.2 *μ*l diluted DAF-FM DA was add into 200 *μ*l single-cell suspensions from *in vitro* BM-derived MDSCs that received various treatments. These cells were incubated in cell incubator at 37°C for 20 minutes. The cells were washed three times with PBS (pH 7.4). After resuspending with 200 *μ*l PBS, the cells were stained with 2 *μ*l Gr-1 and 2 *μ*l CD11b monoclonal antibodies on ice for 15 min. Then, cells gated on CD11b^+^Gr-1^+^ were analyzed for DAF-FM DA mean fluorescence intensity (MFI) on Beckman Coulter CytoFLEX flow cytometry and analyzed with FlowJo software (version 10, TreeStar).

### 2.11. In Vivo Experiments

All experiments with mice were preapproved by the Animal Care and Use Committee of Shenzhen Institutes of Advanced Technology, Chinese Academy of Sciences, under the protocol SIAT-IACUC-20211129-YYS-DBYWZX-YDH-A0802-02. C57BL/6 (6-8-week-old females) were obtained from Guangdong Province Animal Care Facilities and were maintained in the Animal Facilities of Shenzhen Institutes of Advanced Technology, Chinese Academy of Sciences, under pathogen-free conditions. To establish subcutaneous (s.c.) tumors in C57BL/6 mice, 1 × 10^6^ B16F10 or 1 × 10^7^ Hepa 1-6 cells were injected s.c. into the mice. Tumor size and body weights of the mice were recorded every other day starting from the point when tumor growth was palpable (day 5 of Hepa1-6 tumor model or day 9 of B16F10 tumor model) to about 3 weeks or more before mice were sacrificed. Tumor volume was measured using a digital caliper and calculated by using the following formula: [(large diameter) × (small diameter)^2^/2]. To evaluate if targeting inhibition of MDSCs in mice by ART could block tumor progress, C57BL/6 mice were treated intraperitoneally (i.p.) with either DMSO or different doses of ART (12.5, 25, 50, and 100 mg/kg) once every day starting from day 5 of Hepa1-6 tumor model or day 9 of B16F10 tumor model. For the combination assays of ART therapy targeting MDSCs with anti-PD-L1 immunotherapy, we treated with 50 mg/kg ART daily while administrated 10 mg/kg anti-PD-L1 antibodies once every three days, starting from days 9 and 5 following injection of B16F10 or Hepa 1-6 cells, respectively.

### 2.12. Immunohistochemistry

Hepa 1-6 tumors were fixed in 10% neutral buffered formalin, embedded in paraffin, sectioned, and stained with ARG1 (R&D), iNOS (Cell Signaling Technology), Gr-1 (Biolegend), and F4/80 (Biolegend) antibodies. The relative IHC scores were determined by multiplying the staining intensity with the frequency of positive cells. The staining intensity was scored as follows: 0, negative; 1, weak; 2, moderate; and 3, strong. The frequency of positive cells was defined as follows: 0, less than 5%; 1, 5% to 25%; 2, 26% to 50%; 3, 51% to 75%; and 4, greater than 75%.

### 2.13. Statistical Analyses

Statistical analyses were performed using Prism 8.4.2 software (GraphPad Software, CA, USA). All data are presented as mean ± standard error of the mean (SEM), and *P* < 0.05 was considered significant. Each experiment was conducted at least three times unless otherwise indicated. Data analysis was performed by either Student's *t* test, one-way, or two-way ANOVA with Tukey's posttest. In figures, asterisks were used as follows: ∗, *P* ≤ 0.05; ∗∗, *P* ≤ 0.01; ∗∗∗, *P* ≤ 0.001; and ∗∗∗∗, *P* ≤ 0.0001.

## 3. Results

### 3.1. ART Promotes MDSC Apoptosis and Inhibits the Accumulation and Immunosuppressive Function of MDSCs

**Figure 1:**
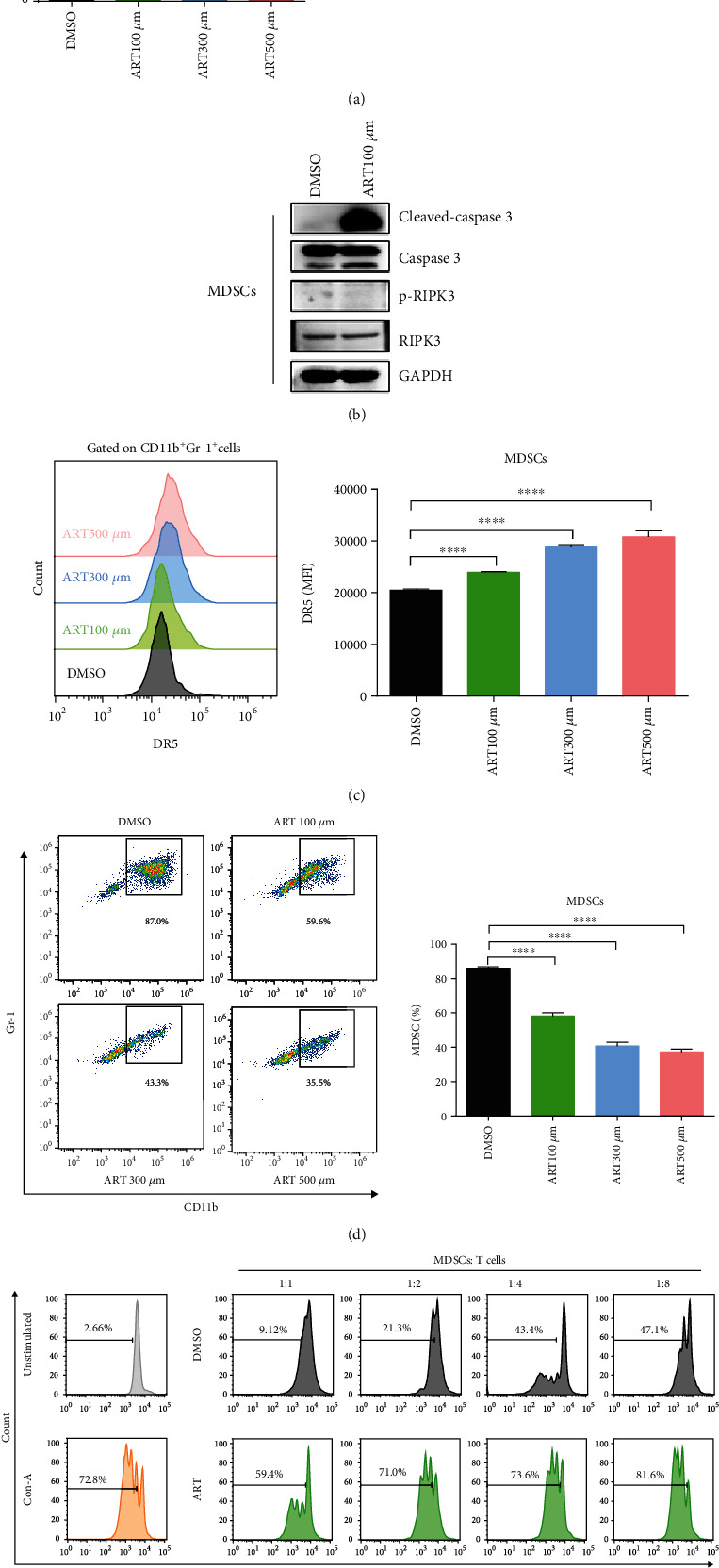
ART promotes MDSC apoptosis and inhibits the accumulation and immunosuppressive function of MDSCs. (a–e) Bone marrow (BM) cells isolated from wild-type C57BL/6 mice were cultured with GM-CSF and IL-6 for 3 days to generate *in vitro* BM-derived MDSCs and then were treated with different concentrations of ART (100 *μ*M, 300 *μ*M, and 500 *μ*M) for another 12 hours, and the solvent DMSO was used as the control. (a) The apoptosis levels of CD11b^+^Gr-1^+^ MDSCs were detected by flow cytometrical analysis. (b) The cleaved-caspase3, caspase3, p-RIPK3, and RIPK3 of CD11b^+^ Gr-1^+^ MDSCs were detected by western blotting. (c) The DR5 mean fluorescence intensity of CD11b^+^ Gr-1^+^ MDSCs was detected by flow cytometrical analysis. (d) The proportion of CD11b^+^ Gr-1^+^ MDSCs was detected by flow cytometric analysis. (e) Flow cytometry to purify CD11b^+^Gr-1^+^ cells from *in vitro* 100 *μ*M ART-treated BM-derived MDSCs and further cocultured CD11b^+^Gr-1^+^ MDSCs with Con A-stimulated CD3^+^ T cells isolated from the spleens of wild-type C57BL/6 at the 1 : 1, 1 : 2, 1 : 4, and 1 : 8 ratios to detect the percentages of proliferation T cells as tested by CFSE fluorescence. Data are means ± SEM and are from a representative experiment of three (a–e) independent experiments. Unpaired Student's _t_ test for (a)–(e). ^∗^*P* < 0.05, ^∗∗∗^*P* < 0.001, and ^∗∗∗∗^*P* < 0.0001.

To investigate if ART can inhibit MDSC accumulation and immunosuppressive function, bone marrow (BM) cells isolated from wild-type C57BL/6 mice were cultured with GM-CSF and IL-6 for 3 days to generate MDSCs *in vitro*. These BM-derived MDSCs were then treated with different concentrations of ART (100 *μ*M, 300 *μ*M, and 500 *μ*M) for 12 hours, and the solvent DMSO was used as control. The apoptosis levels of CD11b^+^Gr-1^+^ MDSCs were detected by flow cytometry (Figure 1(a) and S1A). The results showed that the percentages of Annexin V^+^ apoptosis cells in ART-induced MDSCs were increased in a concentration-dependent manner compared to DMSO-treated group (Figure 1(a)). In addition, ART induced cleaved-caspase 3-dependent apoptosis of MDSCs, but not phosphorylated RIPK3-dependent necroptosis compared to DMSO-treated group (Figure 1(b)). The death receptor 5 (DR5) expression in ART-induced MDSCs was also upregulated in a concentration-dependent manner compared to DMSO-treated group (Figure 1(c) and S1B). Next, we further examined whether ART could affect MDSC generation *in vitro*. We observed that the proportions of CD11b^+^Gr-1^+^ MDSCs were gradually decreased in ART concentration-dependent manner compared to DMSO-treated group (Figure 1(d) and S1C). Above data suggested that 100 *μ*M ART could significantly induce MDSC apoptosis (over 50%) and dramatically reduce MDSC generation (over 40%) compared to DMSO-treated group (below 10% and 13%, respectively) (Figures 1(a) and 1(d)). To further study whether ART could affect the immunosuppressive function of MDSCs, a CFSE-fluorescence-labeled T cell proliferation assay was introduced. To this end, CD3^+^ T cells were isolated from the spleens of wild-type C57BL/6 mice and labeled with CFSE. CD11b^+^Gr-1^+^ cells were sorted by flow cytometry from 100 *μ*M ART-treated BM-derived MDSCs and then cocultured with Con A-stimulated CFSE-labeled CD3^+^ T cells at the ratios of 1 : 1, 1 : 2, 1 : 4, and 1 : 8. The proliferated T cells indicated by CFSE-low proportions were then be detected by flow cytometry. Interestingly, compared to DMSO-treated MDSCs that markedly inhibited T cell proliferation in a ratio-dependent manner, ART-treated MDSCs barely influenced T cell proliferation that is comparable to Con A-stimulated CD3^+^ T cell controls (Figure 1(e) and S1D). In order to distinguish effects of ART on viability and immunosuppression of MDSCs, we checked MDSC-death kinetic in these coculture systems. We found that the apoptosis levels of ART-derived MDSCs during coculture with CD3^+^ T cells at the 1 : 1 ratio were similar to the one of ART-treated MDSCs only. However, the apoptosis levels of ART-derived MDSCs were increased in a T cell ratio-dependent manner compared to the DMSO-treated group. In summary, these data suggested that ART-treated MDSCs had the same viability in the coculture with T cells and T cells further induced the apoptosis of ART-derived MDSCs during coculture at the 1 : 2, 1 : 4, and 1 : 8 ratios (Fig. S1E). These findings indicate that ART can block the accumulation and immune-suppressive function of MDSCs *in vitro*.

### 3.2. ART Promotes to Polarize MDSCs from M2-Like Protumoral Phenotype towards M1-Like Antitumoral One

**Figure 2:**
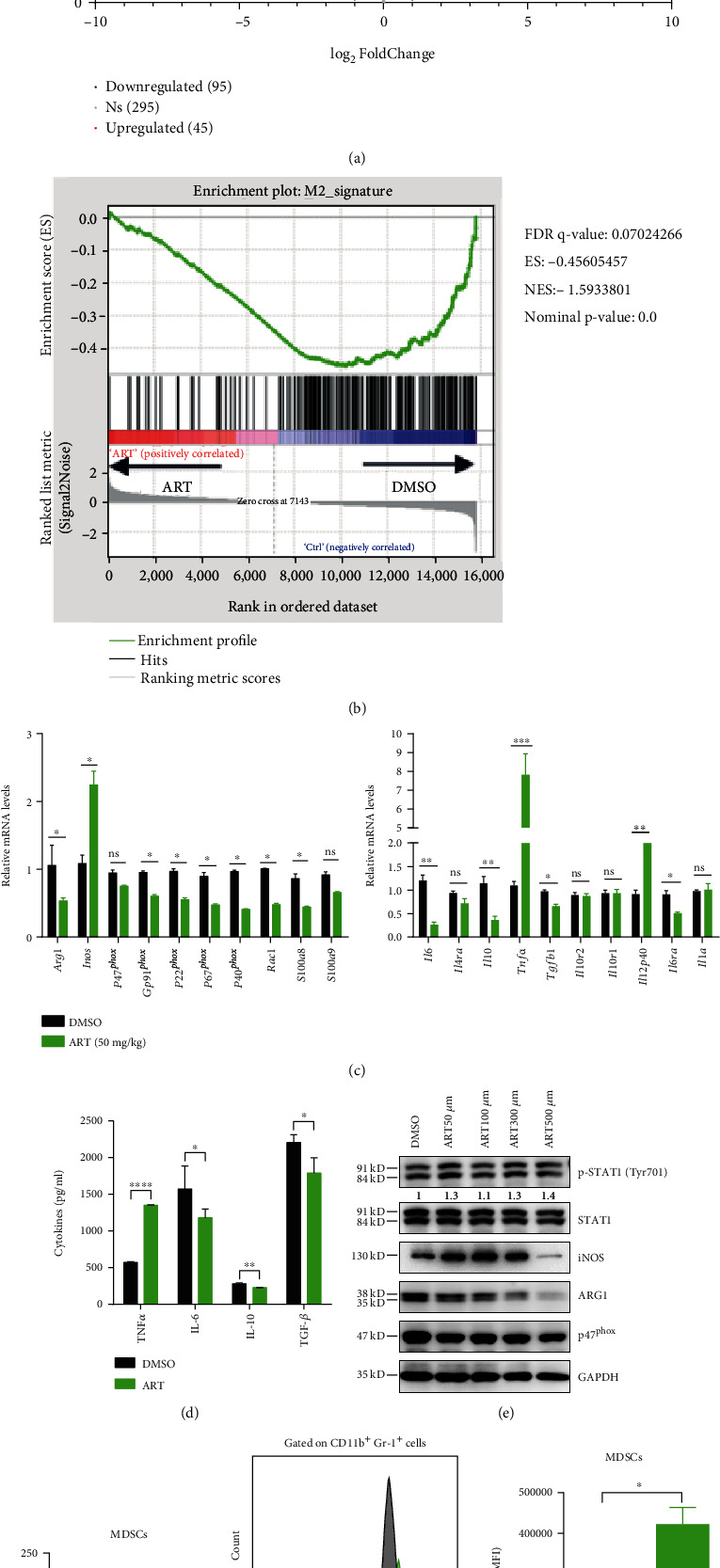
ART promotes to polarize MDSCs from M2-like protumoral phenotype towards M1-like antitumoral one. (a–g) Purified CD11b^+^Gr-1^+^ MDSCs from the tumors of B16F10 melanoma cell-bearing C57BL/6 mice treated with DMSO for 13 days or ART (50 mg/kg) for 18 days (both groups had the similar tumor volume ~1000 mm^3^). (a and b) Performed RNA sequencing (RNA-seq) and gene set enrichment analysis (GSEA) and (c) the expressions of M1 and M2 signature genes were detected by qPCR in tumor MDSCs. (d) The cytokines IL-6, IL-10, TNF-*α*, and TGF-*β* were detected by ELISA. (e) The expressions of p-STAT1 (Tyr701), STAT1, iNOS, ARG1, and p47^phox^ protein were detected by western blot. (f and g) Detected arginase activity by biochemical assays and nitric oxide content by DAF-FM DA fluorescence using flow cytometry analysis. Data are means ± SEM and are from a representative experiment of three (f and g) or from two (c) independent experiments. Unpaired Student's *t* test for (c) and (d) and (f) and (g). ^∗^*P* < 0.05, ^∗∗^*P* < 0.01, ^∗∗∗^*P* < 0.001, and ^∗∗∗∗^*P* < 0.0001. ns: not significant.

In order to determine whether ART controlled the gene expression patterns of MDSCs, we purified CD11b^+^Gr-1^+^ MDSCs from the tumors of B16F10 melanoma cells-bearing C57BL/6 mice. These MDSCs were then treated with DMSO for 13 days or ART (50 mg/kg) for 18 days (both groups had the similar tumor volume ~1000 mm^3^) and applied to RNA sequencing (RNA-seq), gene set enrichment analysis (GSEA), and RT-qPCR analysis. We found that ART downregulated 2861 genes and upregulated 1599 genes in MDSCs compared to DMSO control (Fig. S2A). Interestingly, ART downregulated 95 M2-like immunosuppressive signature genes (such as *Arg1*, *Cd36*, *Gab1*, *Bcl2*, *Irf4*, *Slc25a15*, *Slc27a3*, *Slc6a8*, *Slc16a13*, and *Slc38a4*) while upregulated 45 M1-like immune stimulatory signature genes (such as *Slc17a6*, *Slc52a3*, *Slc36a3*, *Slc22a18*, *Slc1a4*, *Cxcr3*, *Slc24a3*, *Slc34a2*, and *Slc12a7*) and had no change in the additional 295 M1-like or M2-like signature genes in MDSCs [41](Figure 2(a)). GSEA analysis showed that downregulated genes in ART-treated MDSCs were enriched for M2 anti-inflammatory signature (Figure 2(b)). By RT-qPCR, we further confirmed the decrease of a number of M2 immunosuppressive signature genes (including *Arg1*, *Gp91^phox^*, *P22^phox^*, *P67^phox^*, *P40^phox^*, *Rac1*, *S100a8*, *Il6*, *Il10*, *Tgfb1*, and *Il6ra*) and the increase of a number of M1 immune stimulatory signature genes (including *Inos*, *Il12p40*, and *Tnfa*) in ART-treated MDSCs compared with those in DMSO-treated MDSCs. There was no change in other M1 signature gene *Il1a* and other M2 signature genes (including *P47^phox^*, *S100a9*, *Il4ra*, *Il10r2*, and *Il10r1*) of ART-treated MDSCs compared to those of DMSO-treated MDSCs (Figure 2(c)). By cytokine detection, we found that ART upregulated M1-like signature cytokine TNF-*α* and downregulated M2 signature cytokines IL-6, IL-10, and TGF-*β* (Figure 2(d)). By western blot analysis, we also found that ART upregulated iNOS production from MDSCs in a concentration-dependent and STAT1 phosphorylation independent manner compared to DMSO. The optimal treatment concentration of ART that induced the maximal iNOS protein production in MDSCs was 100 *μ*M concentration (Figure 2(e)). However, ART downregulated ARG1 level of MDSCs in a concentration-dependent manner (Figure 2(e)). Moreover, ART had no significant effect on p47^phox^ protein expression of MDSCs as seen in *P47^phox^* mRNA expression of MDSCs (Figure 2(e)). Finally, we further detected arginase activity by biochemical assays and nitric oxide content by DAF-FM DA fluorescence using flow cytometry analysis. The results showed that ART remarkably inhibited arginase activity but increased nitric oxide content of MDSCs compared with DMSO (Figures 2(f) and 2(g) and S2B). These results suggest that ART converted MDSCs from an immunosuppressive phenotype to a stimulatory one through inhibition of M2 signature while promotion of M1 signature gene expression patterns.

### 3.3. ART Controls the Functional Polarization of MDSCs through PI3K/AKT, mTOR, and MAPK Pathways

**Figure 3:**
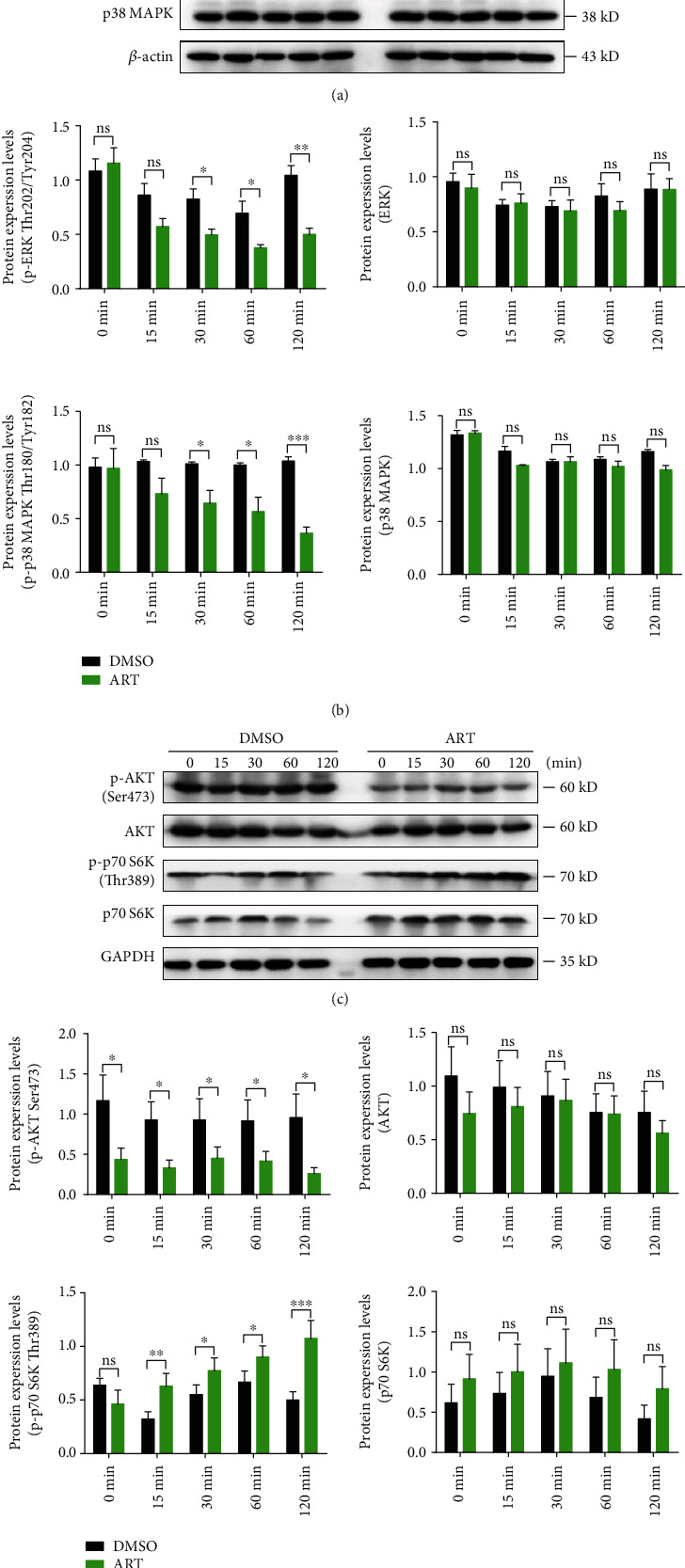
ART controls the functional polarization of MDSCs through PI3K/AKT, mTOR, and MAPK pathways. (a and c) Purified CD11b^+^Gr-1^+^ MDSCs from the tumors of B16F10-bearing C57BL/6 mice for 13 days and then treated MDSCs with DMSO or ART (100 *μ*M) *in vitro* for different time points. (a) Western blot was used to detect MAPK signal pathway-related proteins p-ERK (Thr202/Tyr204), ERK, p-p38 MAPK (Thr180/Tyr182), and p38 MAPK. (b) Gray value statistics. (c) PI3K/AKT and mTOR signaling pathway-related proteins p-AKT (ser473), AKT, p-p70 S6K (Thr389), and p70 S6K were detected by western blot. (d) Gray value statistics. Data are means ± SEM and are from a representative experiment of three (a and c). Unpaired Student's *t* test for (b) and (d). ^∗^*P* < 0.05, ^∗∗^*P* < 0.01, and ^∗∗∗^*P* < 0.001. ns: not significant.

PI3K/AKT, mTOR, and MAPK signaling pathways play important roles in MDSC functional polarization in tumor [24, 42]. In order to further gain insight into whether ART controls MDSC functional polarization through these signaling pathways, we purified CD11b^+^Gr-1^+^ MDSCs from the tumors of B16F10-bearing C57BL/6 mice and then treated with DMSO or ART (100 *μ*M). We first determined the activation of two main MAPK components, ERK and p38 MAPK. Our western blot results showed that ART-treated MDSCs had significantly lowered ERK Thr202/Tyr204 or p38 MAPK Thr180/Tyr182 phosphorylation levels, starting from 30 minutes to 120 minutes compared to DMSO-treated MDSCs. In comparison, the expression levels of unphosphorylated ERK or p38 MAPK proteins between both MDSCs at all time points remained largely unchanged (Figures 3(a) and 3(b)). Next, we further tested PI3K/AKT and mTOR signals in ART-treated MDSCs. The expression levels of phosphorylated AKT Ser473 of ART-treated MDSCs markedly decreased, starting from just a few seconds (labeled “0 min” time point) to “120 min” time point compared with that of DMSO-treated MDSCs, whereas there was no significant change in the expression levels of unphosphorylated AKT of both MDSCs at all time points (Figures 3(c) and 3(d)). However, we found elevated levels of p70 S6K Thr389 phosphorylation protein, one of the major mTOR activation signal proteins, in ART-treated MDSCs starting from 15 minutes to 120 minutes compared to DMSO-treated MDSCs. In parallel, there was no significant difference in the expression levels of unphosphorylated p70 S6K of both MDSCs at all time points (Figures 3(c) and 3(d)). These data indicate that ART controls MDSC functional polarization through activating p70 S6K mTOR and inhibiting PI3K/AKT and MAPK signaling pathways.

### 3.4. Targeting MDSCs by ART Reduces Tumor Growth in Two Mouse Tumor Models

**Figure 4:**
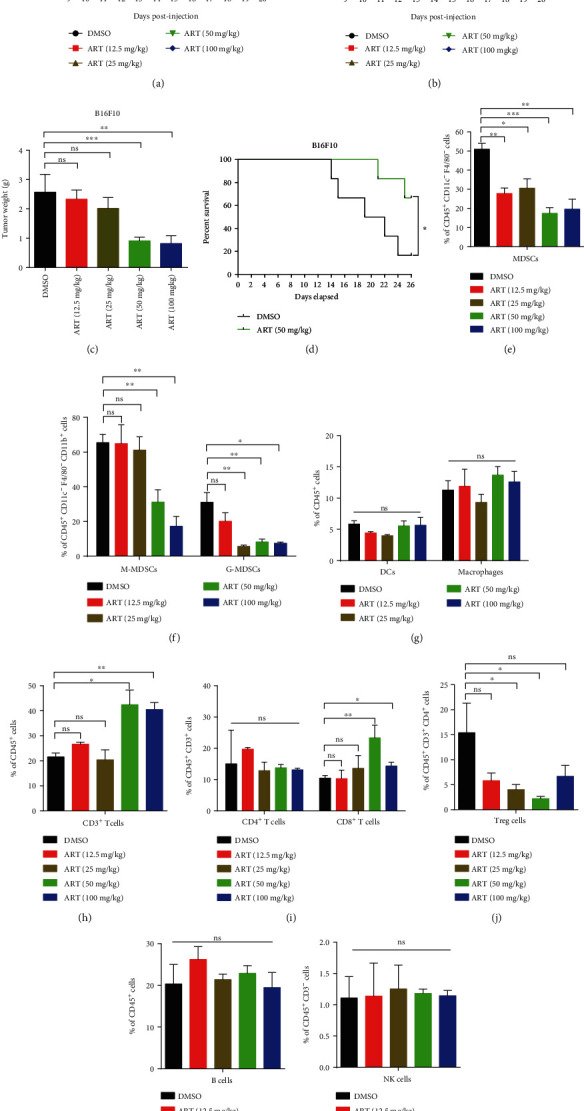
Targeting MDSCs by ART reduces tumor growth in two mouse tumor models. (a–c) C57BL/6 mice injected s.c. on day 0 with B16F10 melanoma cells and received subsequent i.p. injections of either DMSO or different doses of ART (12.5, 25, 50, and 100 mg/kg) once every day starting from day 9 of B16F10 tumor model. (a) Tumor growth curve, (b) mice weight curve, and (c) tumor weight recorded from B16F10 tumor-bearing mice (*n* = 5 mice per group). (d) Survival curve of DMSO and ART 50 mg/kg-treated B16F10 tumor-bearing mice (*n* = 5 mice per group). (e–l) The proportions of immune cells in tumor tissues: MDSCs, M-MDSCs, G-MDSCs, DCs, macrophages, CD3^+^ T cells, CD4^+^ T cells, CD8^+^ T cells, Treg cells, B cells, and NK cells were detected by flow cytometry. MDSCs were CD45^+^CD11c^−^F4/80^−^CD11b^+^Gr-1^+^ cells; M-MDSCs were CD45^+^CD11c^−^F4/80^−^CD11b^+^Ly6G^−^Ly6C^high^ cells; G-MDSCs were CD45^+^ -CD11c^−^F4/80^−^CD11b^+^Ly6G^+^Ly6C^low/int^ cells; DCs were CD45^+^F4/80^−^CD11c^+^ cells; macrophages were CD45^+^CD11c^−^F4/80^+^ cells; CD3^+^ T cells were CD45^+^CD3^+^ cells; CD4^+^ T cells were CD45^+^CD3^+^CD4^+^CD8^−^ cells; CD8^+^ T cells were CD45^+^CD3^+^CD4^−^CD8^+^ cells; Treg cells were CD45^+^CD3^+^CD4^+^CD25^+^Foxp3^+^ cells; B cells were CD45^+^CD19^+^ cells; and NK cells were CD45^+^CD3^−^CD4^−^NK1.1^+^ cells. Data are means ± SEM and are from a representative experiment of three (a–c and e–l). Unpaired Student's *t* test for (a)–(c) and (e)–(l). Two-sided log-rank test for (d). ^∗^*P* < 0.05, ^∗∗^*P* < 0.01, and ^∗∗∗^*P* < 0.001. ns: not significant.

Above data have shown that ART could inhibit MDSC development and immunosuppressive function via regulating its functional polarization. In order to further evaluate if targeting MDSCs by ART could suppress tumor progress, we compared tumor growth kinetics of B16F10 melanoma and Hepa1-6 hepatoma under ART treatment. To this end, C57BL/6 mice were subcutaneously injected by B16F10 or Hepa1-6 cells and then treated by DMSO or ART (at doses of 12.5, 25, 50, and 100 mg/kg) once every day starting at day 5 or day 9 post Hepa1-6 or B16F10 inoculation. We found that consistently decreased tumor volume, tumor weight, and tumor size were observed in ART-treated mice in a dose-dependent manner compared with DMSO-treated mice. However, only 50 mg/kg and 100 mg/kg ART significantly inhibited tumor growth (Figures 4(a) and 4(c) and S4A-C and S4E), and 50 mg/kg ART ameliorated the survival of B16F10 and Hepa 1-6 tumor-bearing mice (Figure 4(d) and S4F). No change in the body weight was observed in ART-treated mice compared to DMSO-treated ones (Figure 4(b) and S4D).

We then asked whether ART treatment affected the distribution of MDSCs. Figs. S3A-C showed the flow cytometry gating strategies for immune cells in tumor tissues. We found that only treatment with 50 mg/kg ART significantly decreased the percentages of MDSCs, M-MDSCs, G-MDSCs, and Treg cells but increased the percentages of CD3^+^ and CD8^+^ T cells in B16F10 and Hepa 1-6-bearing mice. In comparison, the increase of CD4^+^ T cells was only detected in ART-treated Hepa 1-6-bearing mice (Figures 4(e), 4(f), and 4(h)–4(j) and S4G-H and S4J-L). For B16F10 tumor model, treatment with 100 mg/kg ART significantly decreased the percentages of MDSCs, M-MDSCs, and G-MDSCs but increased the percentages of CD3^+^ and CD8^+^ T cells, while there was no change in the percentages of CD4^+^ T cells and Treg cells compared to treatment with DMSO (Figures 4(e), 4(f), and 4(h)–4(j)). However, for Hepa 1-6 tumor model, treatment with 100 mg/kg ART could decrease the percentages of MDSCs, G-MDSCs, and Treg cells but increased the percentages of CD3^+^, CD4^+^, and CD8^+^ T cells, while there was no change in the percentages of M-MDSCs compared to treatment with DMSO (Figs. S4G-H and S4J-L). Interestingly, treatment with 25 mg/kg ART could decrease the percentages of MDSCs, M-MDSCs, G-MDSCs, and Treg cells but did not affect the percentages of CD3^+^, CD4^+^, and CD8^+^ T cells in both tumor models (Figures 4(e), 4(f), and 4(h)–4(j) and S4G-H and S4J-L). Besides, treatment with 12.5 mg/kg ART could decrease the percentages of MDSCs but did no change in the percentages of M-MDSCs, G-MDSCs, Treg cells, CD3^+^, CD4^+^, and CD8^+^ T cells in both tumor models (Figures 4(e), 4(f), and 4(h)–4(j) and S4G-H and S4J-L). In addition, treatment with all the concentrations of ART did not alter the percentages of DCs, macrophages, and B and NK cells in B16F10 and Hepa 1-6-bearing mice compared to treatment with DMSO (Figures 4(g), 4(k), and 4(l) and S4I and S4M-N). To further illustrate the effect of ART on the immune landscape of in situ tumor microenvironment, we used immunohistochemistry to detect the M1 marker iNOS and M2 marker ARG1 of MDSCs (Gr-1^+^ cells) and macrophages (F4/80^+^ cells). The results showed that ART obviously decreased M2 MDSCs and macrophages but increased M1 MDSCs and macrophage entry into the tumor sites (Figs. S4O-P). These results indicate that ART treatment in mice leads to the inhibition of MDSCs while increasing CD3^+^ T cell and CD8^+^ T cell infiltration to the tumor sites and thus reduces tumor growth.

### 3.5. Targeting MDSCs via ART Therapy Significantly Enhances the Efficacy of Anti-PD-L1 Immunotherapy in Tumor-Bearing Mice

**Figure 5:**
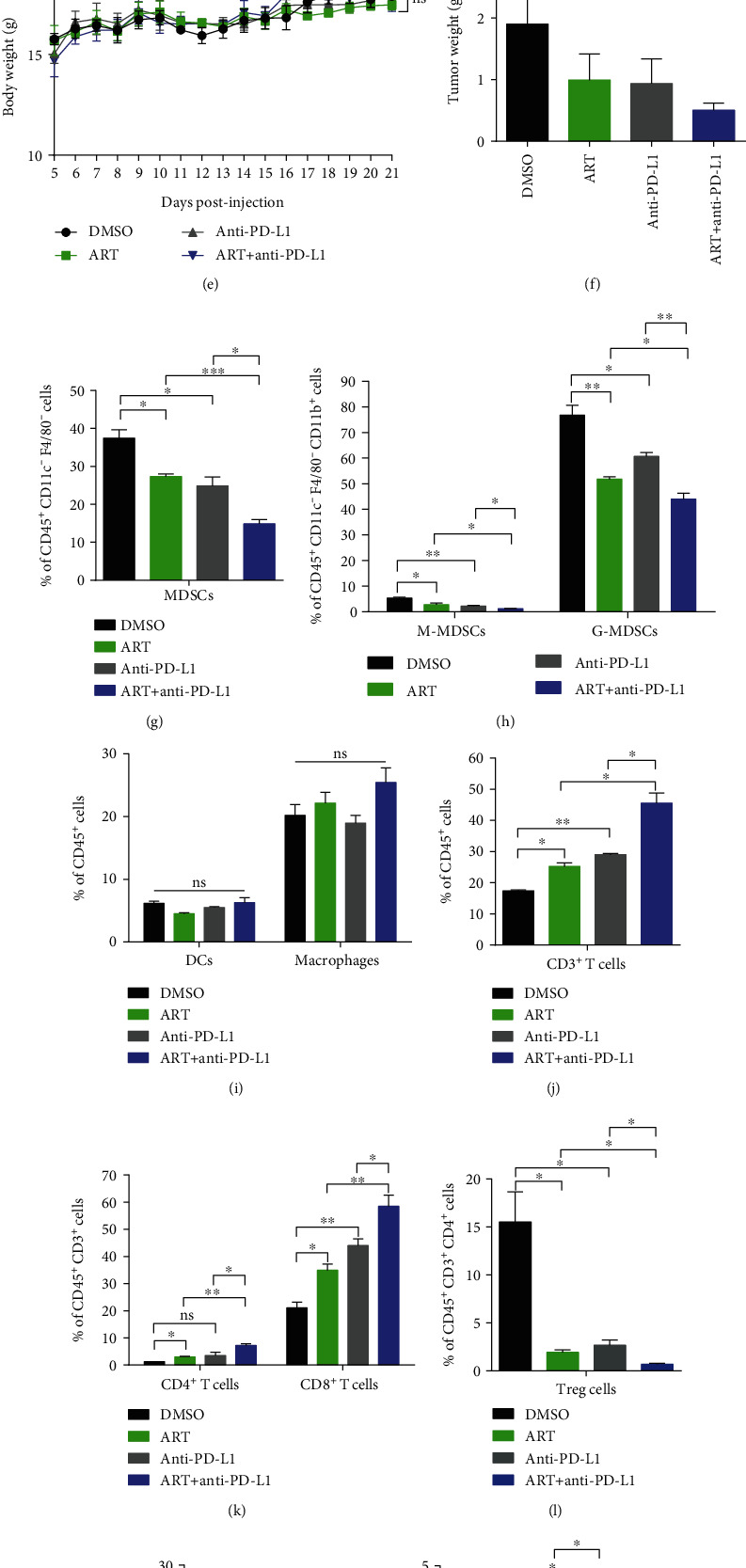
Targeting MDSCs via ART therapy significantly enhances the efficacy of anti-PD-L1 immunotherapy in tumor-bearing mice. (a–o) In B16F10 or Hepa 1-6-bearing mice, we treated with 50 mg/kg ART daily while administrated 10 mg/kg anti-PD-L1 antibodies once every three days, starting from days 9 and 5 following injection of B16F10 or Hepa 1-6 cells, respectively (*n* = 5 mice per group). (a and d) Tumor growth curves, (b and e) mice weight growth curves, and (c and f) tumor weights recorded from B16F10 or Hepa 1-6 tumor-bearing mice. (g–n) The proportions of immune cells in tumor tissues of Hepa 1-6 tumor-bearing mice: MDSCs, M-MDSCs, G-MDSCs, DCs, Macrophages, CD3^+^ T cells, CD4^+^ T cells, CD8^+^ T cells, Treg cells, B cells, and NK cells were detected by flow cytometry. (o) Tumor MDSCs isolated from Hepa 1-6-bearing mice were treated with the combination therapy of ART and anti-PD-L1 antibodies cocultured at 1 : 2 ratio with CFSE-labeled spleen CD3^+^ T cells purified from wild-type C57BL/6 mice activated with Con A (5 *μ*g/ml). The cells were cultured for 3 days and stained with CD3 antibody, and T cell proliferation was analyzed by flow cytometry. Data are means ± SEM and are from a representative experiment of three (a–n) or from two (o) independent experiments. Unpaired Student's *t* test for (a)–(n). ^∗^*P* < 0.05, ^∗∗^*P* < 0.01, ^∗∗∗^*P* < 0.001, and ^∗∗∗∗^*P* < 0.0001. ns: not significant.

Next, we explored whether the combination of targeting MDSCs via ART therapy with anti-PD-L1 immunotherapy could further enhance antitumor effect in tumor-bearing mice. We therefore treated B16F10 or Hepa 1-6-bearing mice with 50 mg/kg ART (this concentration was enough for inhibition of both MDSCs subsets) daily, with or without administrating 10 mg/kg anti-PD-L1 antibodies every three days, starting as described above. We noted that either ART or anti-PD-L1 antibody alone had an antitumor effect in B16F10 or Hepa 1-6-bearing mice compared to DMSO (Figures 5(a) and 5(d)). Interestingly, the combined ART and anti-PD-L1 antibody dramatically delayed tumor progression and reduced tumor weight and size compared with the monotherapy (Figures 5(a), 5(c), 5(d), and 5(f) and S5A). No difference in the body weight of all mice with monotherapy or combination therapy was observed (Figures 5(b) and 5(e)).

We further determined whether the combined ART with anti-PD-L1 immunotherapy could affect the accumulation and function of MDSCs. By flow cytometry, we observed that single anti-PD-L1 antibody could reduce the percentages of MDSCs, M-MDSCs, G-MDSCs, and Treg cells, while it raised the percentages of CD3^+^ T cells and CD8^+^ T cells in Hepa 1-6-bearing mice compared to treatment with DMSO (Figures 5(g), 5(h), and 5(j)–5(l)). Surprisingly, the combination of ART and anti-PD-L1 antibody treatment further decreased the percentages of MDSCs, M-MDSCs, G-MDSCs, and Treg cells but additionally increased the percentages of CD3^+^ T cells, CD4^+^ T cells, CD8^+^ T cells, and NK cells in Hepa 1-6-bearing mice compared with the anti-PD-L1 monotherapy (Figures 5(g), 5(h), 5(j)–5(l), and 5(n)). Nevertheless, neither the anti-PD-L1 monotherapy nor the combinatory therapy varied the percentages of DCs, macrophages, and B cells in Hepa 1-6-bearing mice (Figures 5(i) and 5(m)). Considering that the significant difference in tumor volume of mice between the anti-PD-L1 monotherapy and the combinatory therapy could affect the immunological study, we isolated tumor tissues in the similar tumor volume ~1000 mm^3^ in the different time: DMSO group on day 12, ART or anti-PD-L1 monotherapy group on day 15, and the combination therapy group on day 21. Similarly, the combination of ART and anti-PD-L1 antibody treatment further decreased the percentages of MDSCs, G-MDSCs, and Treg cells but additionally increased the percentages of CD3^+^ T cells and CD8^+^ T cells in Hepa 1-6-bearing mice compared with the anti-PD-L1 monotherapy (Figs. S5B-C and S5E-G). Nevertheless, the combination therapy of ART and anti-PD-L1 antibody did not vary the percentages of M-MDSCs, DCs, macrophages, CD4^+^ T cells, B cells, and NK cells in Hepa 1-6-bearing mice (Figs. S5C-D, F and S5H-I). Although tumor-derived MDSCs from anti-PD-L1 treated Hepa 1-6-bearing mice showed the capacity to inhibit T cell proliferation as those from DMSO-treated mice, tumor-derived MDSCs from the combination therapy group had a further reduced capacity to block T cell proliferation compared to ART therapy group (Figure 5(o)). These results suggest that ART therapy inhibiting MDSC accumulation and function further enhanced the efficacy of anti-PD-L1 immunotherapy in tumor-bearing mice through enhancement of CD3^+^ T cell migration and proliferation.

## 4. Discussion

Here, we report that ART may potently regulate MDSCs by controlling the switch between protumoral and antitumoral gene expression pattern. ART-treated MDSCs lose the immunosuppressive function and ART-treated mice reduced tumor progression.

ART, as an old antimalarial drug, has shown its antitumor activities through direct induction of tumor cell death by releasing excessive ROS and indirect regulation of immune cell responses against tumor [43, 44]. Previous reports showed that ART could induce tumor cell apoptosis and ferroptosis by ferrous iron-dependent ROS-triggered ER stress [45]. In addition, ART promotes the tumor cytotoxicity of T, *γδ*T, and NK cells but lowers Treg and MDSC frequencies in tumor-bearing mice [37, 46]. In our study, ART treatment decreases MDSC accumulation through DR5-induced apoptosis in MDSCs. More importantly, ART supplement almost completely prevents the immunosuppressive function of MDSCs, as shown by *in vitro* ART-treated MDSC coculture assays and *in vivo* mouse models, in which ART treatment promotes T cell tumor migration and thus inhibits tumor growth in mice (Figures 1(a)–1(e)). Interestingly, ART-treated MDSCs boost relatively higher proliferation of Con A-stimulated CD3^+^ T cells at 1 : 8 ratio coculture system (Figure 1(e)), suggesting that ART may promote MDSCs transpolarizing to immunostimulatory cells. These results suggest that ART can reverse the immunosuppressive microenvironment by targeting inhibition of MDSC accumulation and immune-suppressive activities.

ART and its derivatives may regulate immune cells by affecting different signaling pathways in cancer treatment [38]. ART promotes CD8^+^ T cell activation by upregulating T-bet expression and decreases the percentage of Tregs by inhibiting Foxp3 expression in 4T1 breast tumor-bearing mice [47]. ART enhances NK cell cytotoxicity against K562 tumor cells by activating ERK1/2 signaling pathway [48]. Dihydroartemisinin, one of ART derivatives, strengthens *γδ*T cell killing activities against pancreatic cancer cells through increasing intracellular perforin, granzyme B, and IFN-*γ* production signaling pathways [49]. In addition, artesunate, another ART derivative, enables tumor-associated monocytes to repolarize tumoricidal inflammatory monocytes against leukemic cells by inhibition of JAK2/STAT3 pathway [50]. Interestingly, the nanoparticles encapsulated chemotherapeutic drug oxaliplatin, and dihydroartemisinin significantly enhances the tumor antigen uptake and presentation of both dendritic cells and macrophages through MHC I signals in colorectal tumor-bearing mice [51]. Although recent study showed that ART decreases MDSC frequencies in 4T1-bearing mice [47] and improves the efficiency of anti-PD-L1 blockade in T cell lymphoma-bearing mice [39, 40], how ART influences MDSC accumulation, function, and molecular pathways in melanoma or liver tumors and further enhances PD-L1 blockade-mediated tumor immunotherapy remains unknown. In our study, we observed that ART remarkably blocks the accumulation and immunosuppressive function of MDSCs through downregulating MAPK and PI3K/AKT signaling axes but upregulating p70 S6K mTOR pathway (Figures 3(a) and 3(b)). ART-induced signaling pathway switch in tumor MDSCs leads to inhibit M2-like tumor-promoting gene expression profiles such as *Arg1*, *Il10*, *Tgfb1*, *Cd36*, *Gab1*, *Bcl2*, and *Irf4* but triggered M1-like antitumor gene expression patterns like *Inos*, *Tnfa*, *Il12p40*, *Cxcr3*, *Slc1a4*, or *Slc22a18* (Figures 2(a)–2(c)). Notably, iNOS is a known mechanism of MDSC-mediated immunosuppression [18]. Meanwhile, M1 macrophages upregulate the expression of iNOS generating NO from arginine to enhance producing proinflammatory cytokines for immunoactivation [52]. Our results showed that ART-treated MDSCs lose the immunosuppressive function and polarize to immunostimulatory myeloid cells through increasing iNOS expression generating NO for activating T cell function (Figures 1(e) and 2(g)). These results indicate that ART contributes to reprogramme MDSC functional polarization through regulating PI3K/AKT, mTOR, and MAPK pathways.

Our data show that targeting blockade of MDSCs by ART further enhances anti-PD-L1 immunotherapy (Figures 5(a) and 5(d)). Most patients with melanoma and hepatocellular carcinoma initially respond to but later become resistant to anti-PD-L1 tumor immunotherapy [6, 53]. The main reason for this resistance is the presence of tumor immunosuppressive microenvironment which mainly consists of MDSCs, tumor-associated macrophages (TAMs), and Tregs [54]. In our experiments, combinating ART and anti-PD-L1 antibody significantly decrease the percentages of MDSCs and its subsets (M-MDSCs and G-MDSCs), Treg cells without changing the proportions of DCs and macrophages in tumors compared to anti-PD-L1 single therapy (Figures 5(g)–5(i) and 5(l)). Furthermore, the deduction of MDSCs by the combination therapy increased CD3^+^, CD4^+^, and CD8^+^ T cell infiltration to tumor tissues more than anti-PD-L1 single therapy (Figures 5(j) and 5(k)). Moreover, the combination therapy may further improve T cell proliferation (Figure 5(o)). Thus, the combination therapy of targeting MDSCs by ART and anti-PD-L1 antibody produces better therapeutic efficacy than the single anti-PD-L1 tumor immunotherapy.

In summary, here we report that ART controls the functional polarization of MDSCs through PI3K/AKT, mTOR, and MAPK pathways and inhibition of MDSCs by ART may provide a novel therapeutic strategy to enhance anti-PD-L1 cancer immunotherapy.

## Data Availability

The data that support the findings of this study are available from the corresponding author upon reasonable request.

## Supplementary Materials

Supplementary 1 Figure S1: flow cytometry gating strategies for detection apoptosis, DR5 expression of MDSCs, and CFSE staining of T cells. (A–C) Bone marrow (BM) cells isolated from wild-type C57BL/6 mice were cultured with GM-CSF and IL-6 for 3 days to generate *in vitro* BM-derived MDSCs and then were treated with different concentrations of ART (100 *μ*M, 300 *μ*M, and 500 *μ*M) for another 12 hours, and the solvent DMSO was used as the control. (A) The apoptosis levels of CD11b^+^Gr-1^+^ MDSCs were detected by flow cytometrical analysis. (B) The DR5 mean fluorescence intensity of CD11b^+^ Gr-1^+^ MDSCs was detected by flow cytometrical analysis. (C) The proportion of CD11b^+^ Gr-1^+^ MDSCs was detected by flow cytometric analysis. (D) Flow cytometry gating strategy to detect the percentages of proliferation Con A-stimulated live CD3^+^ T cells as tested by CFSE fluorescence. (E) The apoptosis levels of CD11b^+^Gr-1^+^ MDSCs in coculture system were detected by flow cytometrical analysis. ^∗^*P* < 0.05, ^∗∗^*P* < 0.01, ^∗∗∗^*P* < 0.001, and ^∗∗∗∗^*P* < 0.0001. ns: not significant.

Supplementary 2 Figure S2: differential expression of total genes in ART-treated MDSCs and flow cytometry gating strategy for NO detection of MDSCs. (A) Performed RNA sequencing (RNA-seq) of total genes and (B) nitric oxide content by DAF-FM DA fluorescence using flow cytometry analysis in ART-treated MDSCs.

Supplementary 3 Figure S3: flow cytometry plots illustrating the gating strategy used for leukocytes analysis.(A–C) Representative flow cytometry plots showing gating strategy from Figures 4(e)–4(l) and 5(g)–5(n) and S4G-N and S5B-I. (A) Flow cytometry analysis of myeloid cells. (B and C) Flow cytometric analysis of lymphocytes. MDSCs were CD45^+^CD11c^−^F4/80^−^CD11b^+^Gr-1^+^ cells; M-MDSCs were CD45^+^CD11c^−^F4/80^−^CD11b^+^Ly6G^−^Ly6C^high^ cells; G-MDSCs were CD45^+^CD11c^−^F4/80^−^CD11b^+^Ly6G^+^Ly6C^low/int^ cells; DCs were CD45^+^F4/80^−^CD11c^+^ cells; macrophages were CD45^+^CD11c^−^F4/80^+^ cells; CD3^+^ T cells were CD45^+^CD3^+^ cells; CD4^+^ T cells were CD45^+^CD3^+^CD4^+^CD8^−^ cells; CD8^+^ T cells were CD45^+^CD3^+^CD4^−^CD8^+^ cells; Treg cells were CD45^+^CD3^+^CD4^+^CD25^+^Foxp3^+^ cells; B cells were CD45^+^CD19^+^ cells; and NK cells were CD45^+^CD3^−^CD4^−^NK1.1^+^ cells. SSC-A: side scatter-area; FSC-A: forward scatter-area; FSC-H: forward scatter-height.

Supplementary 4 Figure S4: targeting MDSCs by ART reduces tumor growth in two mouse tumor models. (A–P) C57BL/6 mice injected s.c. on day 0 with B16F10 melanoma cells or Hepa 1-6 hepatoma cells received subsequent i.p. injections of either DMSO or different doses of ART (12.5, 25, 50, and 100 mg/kg) once every day starting from day 9 of B16F10 tumor model or day 5 of Hepa 1-6 tumor model (*n* = 5 mice per group). (A and B) Images of tumor tissues excised from B16F10 and Hepa 1-6 tumor-bearing mice on day 20 or day 21, respectively. Scale bars, 10 mm. (C) Tumor growth curve, (D) mice weight curve, and (E) tumor weight of Hepa 1-6 tumor-bearing mice. (F) Survival curve of DMSO and ART 50 mg/kg treated Hepa 1-6 tumor-bearing mice (*n* = 5 mice per group). (G–N) The proportions of immune cells in tumor tissues: MDSCs, M-MDSCs, G-MDSCs, DCs, macrophages, CD3^+^ T cells, CD4^+^ T cells, CD8^+^ T cells, Treg cells, B cells, and NK cells were detected by flow cytometry. (N) Survival curve of B16F10 tumor-bearing mice treated with DMSO and ART (50 mg/kg). (O and P) Representative images (O) of IHC staining and the relative IHC scores (P) of ARG1, iNOS, Gr-1, and F4/80. Scale bars, 50 *μ*m. Data are means ± SEM and are from a representative experiment of three (A–E, G–N, and P). Unpaired Student's *t* test for (A)–(E), (G)–(N), and (P). Two-sided log-rank test for (F). ^∗^*P* < 0.05 and ^∗∗^*P* < 0.01. ns: not significant.

Supplementary 5 Figure S5: targeting MDSCs via ART therapy significantly enhances the efficacy of anti-PD-L1 immunotherapy in tumor-bearing mice. (A–I) C57BL/6 mice injected s.c. on day 0 with Hepa1-6 hepatoma cells and treated with 50 mg/kg ART once every day starting from day 5 while administrated 10 mg/kg anti-PD-L1 antibodies every three days starting from day 5 of Hepa 1-6 tumor model. (A) The image of tumor tissues excised from Hepa 1-6 tumor-bearing mice on day 21. Scale bars, 10 mm. (B–I) Tumor tissues were isolated in the similar tumor volume ~1000 mm^3^: DMSO on day 12, ART or anti-PD-L1 on day 15, and ART+anti-PD-L1 on day 21. The proportions of immune cells in tumor tissues: MDSCs, M-MDSCs, G-MDSCs, DCs, macrophages, CD3^+^ T cells, CD4^+^ T cells, CD8^+^ T cells, Treg cells, B cells, and NK cells were detected by flow cytometry. Data are means ± SEM and are from a representative experiment of three (B–I). Unpaired Student's *t* test for (B)–(I). ^∗^*P* < 0.05, ^∗∗^*P* < 0.01, and ^∗∗∗^*P* < 0.001. ns: not significant.

Supplementary 6 Table S1: list of qPCR primer sequences.
