# Supplementary figures and images for: Targeting Inhibition of Accumulation and Function of Myeloid-Derived Suppressor Cells by Artemisinin via PI3K/AKT, mTOR, and MAPK Pathways Enhances Anti-PD-L1 Immunotherapy in Melanoma and Liver Tumors
Source: J Immunol Res. 2022 Jun 22;2022:2253436. doi: 10.1155/2022/2253436 (PMC9247850; doi:10.1155/2022/2253436)

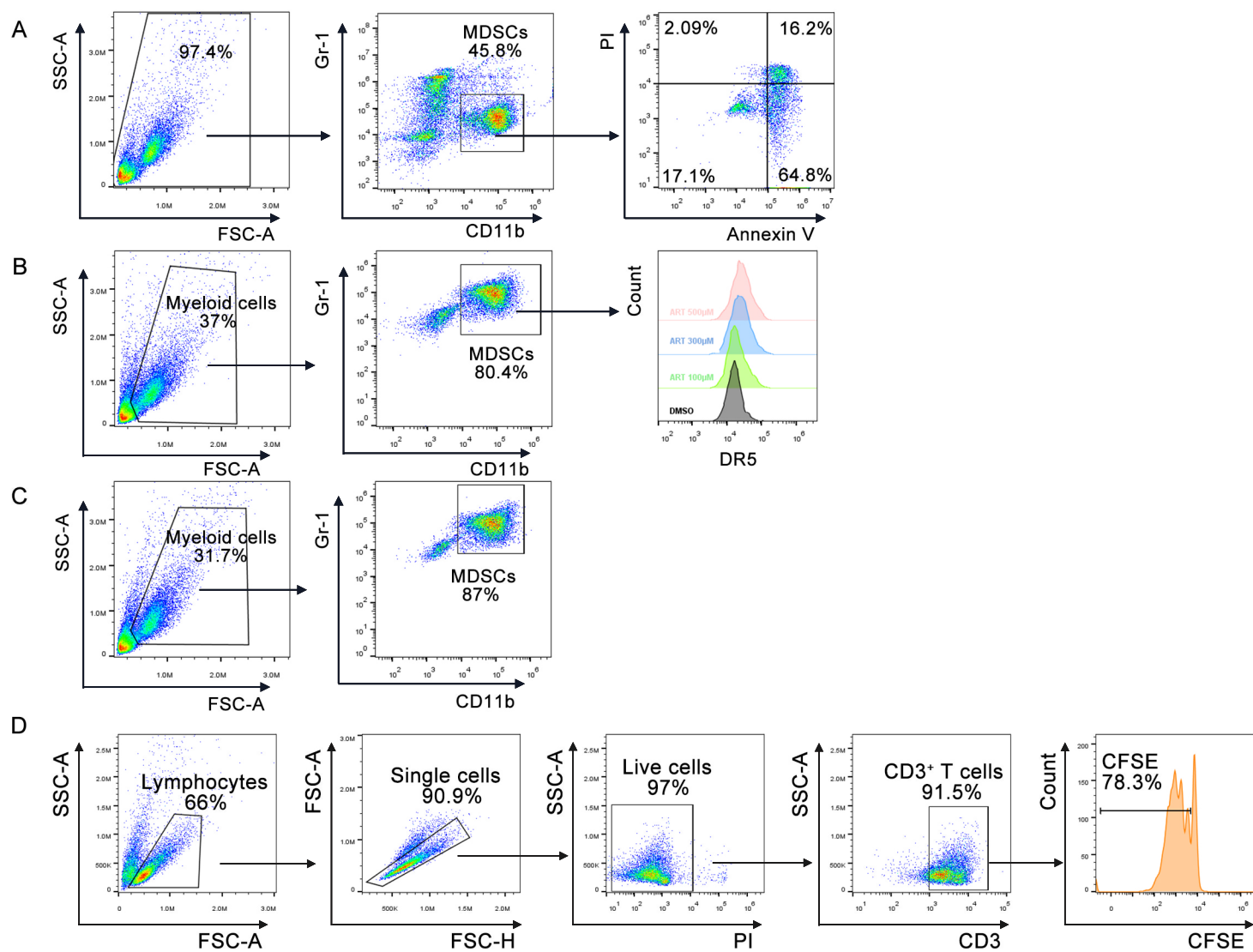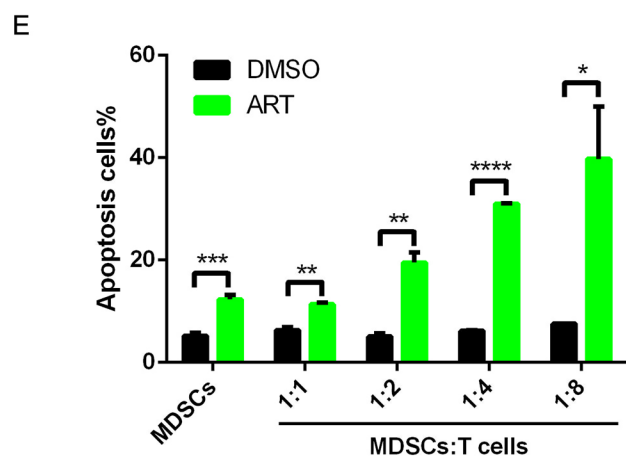

Supplement: Supplementary 1 — Figure S1: flow cytometry gating strategies for detection apoptosis, DR5 expression of MDSCs, and CFSE staining of T cells. (A–C) Bone marrow (BM) cells isolated from wild-type C57BL/6 mice were cultured with GM-CSF and IL-6 for 3 days to generate in vitro BM-derived MDSCs and then were treated with different concentrations of ART (100 μM, 300 μM, and 500 μM) for another 12 hours, and the solvent DMSO was used as the control. (A) The apoptosis levels of CD11b+Gr-1+ MDSCs were detected by flow cytometrical analysis. (B) The DR5 mean fluorescence intensity of CD11b+ Gr-1+ MDSCs was detected by flow cytometrical analysis. (C) The proportion of CD11b+ Gr-1+ MDSCs was detected by flow cytometric analysis. (D) Flow cytometry gating strategy to detect the percentages of proliferation Con A-stimulated live CD3+ T cells as tested by CFSE fluorescence. (E) The apoptosis levels of CD11b+Gr-1+ MDSCs in coculture system were detected by flow cytometrical analysis. ∗P < 0.05, ∗∗P < 0.01, ∗∗∗P < 0.001, and ∗∗∗∗P < 0.0001. ns: not significant. [file 2253436.f1.pdf]

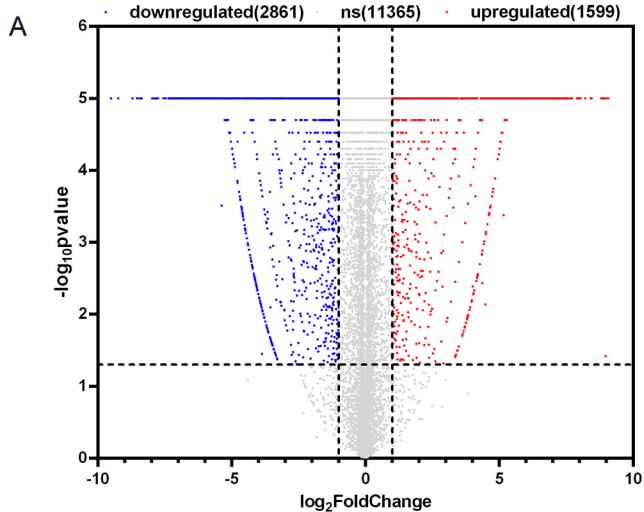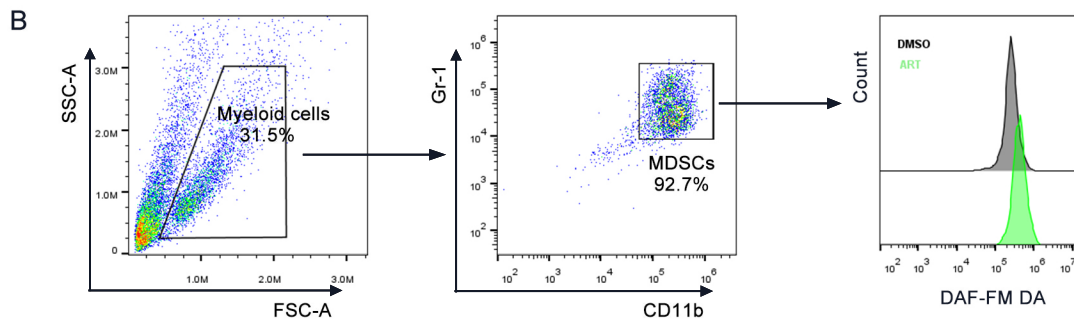

Supplement: Supplementary 2 — Figure S2: differential expression of total genes in ART-treated MDSCs and flow cytometry gating strategy for NO detection of MDSCs. (A) Performed RNA sequencing (RNA-seq) of total genes and (B) nitric oxide content by DAF-FM DA fluorescence using flow cytometry analysis in ART-treated MDSCs. [file 2253436.f2.pdf]

A

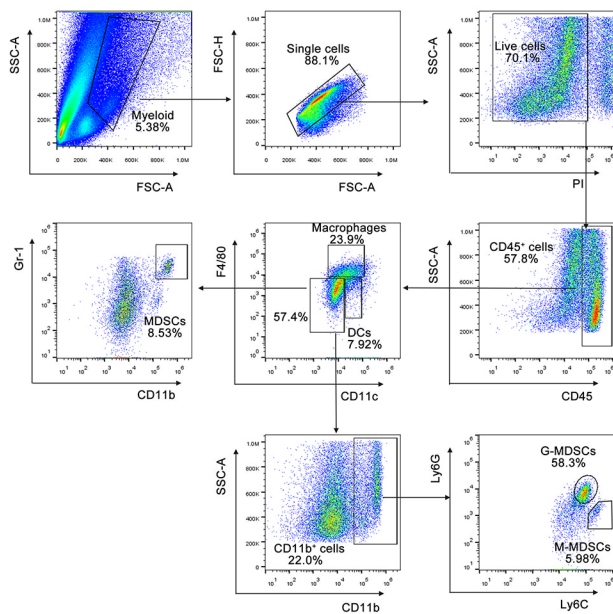

B

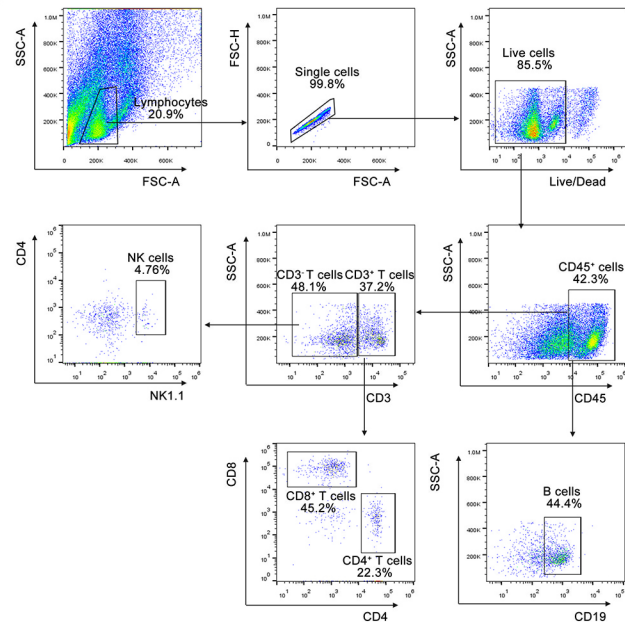

C

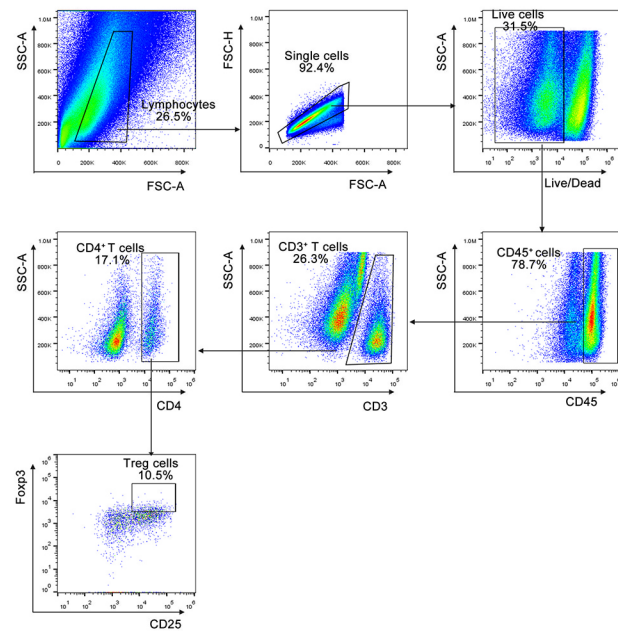

Supplement: Supplementary 3 — Figure S3: flow cytometry plots illustrating the gating strategy used for leukocytes analysis.(A–C) Representative flow cytometry plots showing gating strategy from Figures 4(e)–4(l) and 5(g)–5(n) and S4G-N and S5B-I. (A) Flow cytometry analysis of myeloid cells. (B and C) Flow cytometric analysis of lymphocytes. MDSCs were CD45+CD11c−F4/80−CD11b+Gr-1+ cells; M-MDSCs were CD45+CD11c−F4/80−CD11b+Ly6G−Ly6Chigh cells; G-MDSCs were CD45+CD11c−F4/80−CD11b+Ly6G+Ly6Clow/int cells; DCs were CD45+F4/80−CD11c+ cells; macrophages were CD45+CD11c−F4/80+ cells; CD3+ T cells were CD45+CD3+ cells; CD4+ T cells were CD45+CD3+CD4+CD8− cells; CD8+ T cells were CD45+CD3+CD4−CD8+ cells; Treg cells were CD45+CD3+CD4+CD25+Foxp3+ cells; B cells were CD45+CD19+ cells; and NK cells were CD45+CD3−CD4−NK1.1+ cells. SSC-A: side scatter-area; FSC-A: forward scatter-area; FSC-H: forward scatter-height. [file 2253436.f3.pdf]

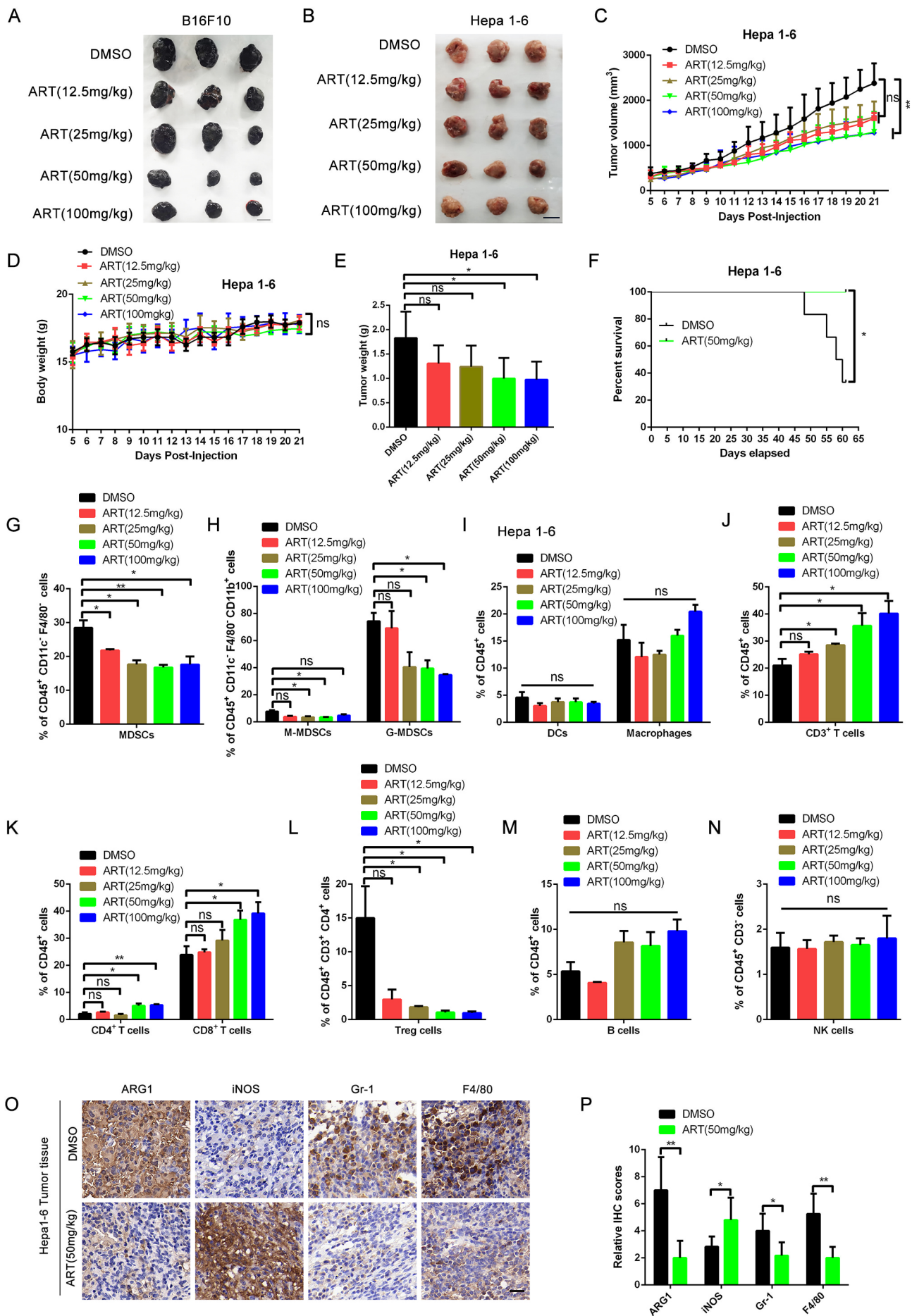

Supplement: Supplementary 4 — Figure S4: targeting MDSCs by ART reduces tumor growth in two mouse tumor models. (A–P) C57BL/6 mice injected s.c. on day 0 with B16F10 melanoma cells or Hepa 1-6 hepatoma cells received subsequent i.p. injections of either DMSO or different doses of ART (12.5, 25, 50, and 100 mg/kg) once every day starting from day 9 of B16F10 tumor model or day 5 of Hepa 1-6 tumor model (n = 5 mice per group). (A and B) Images of tumor tissues excised from B16F10 and Hepa 1-6 tumor-bearing mice on day 20 or day 21, respectively. Scale bars, 10 mm. (C) Tumor growth curve, (D) mice weight curve, and (E) tumor weight of Hepa 1-6 tumor-bearing mice. (F) Survival curve of DMSO and ART 50 mg/kg treated Hepa 1-6 tumor-bearing mice (n = 5 mice per group). (G–N) The proportions of immune cells in tumor tissues: MDSCs, M-MDSCs, G-MDSCs, DCs, macrophages, CD3+ T cells, CD4+ T cells, CD8+ T cells, Treg cells, B cells, and NK cells were detected by flow cytometry. (N) Survival curve of B16F10 tumor-bearing mice treated with DMSO and ART (50 mg/kg). (O and P) Representative images (O) of IHC staining and the relative IHC scores (P) of ARG1, iNOS, Gr-1, and F4/80. Scale bars, 50 μm. Data are means ± SEM and are from a representative experiment of three (A–E, G–N, and P). Unpaired Student's t test for (A)–(E), (G)–(N), and (P). Two-sided log-rank test for (F). ∗P < 0.05 and ∗∗P < 0.01. ns: not significant. [file 2253436.f4.pdf]

A

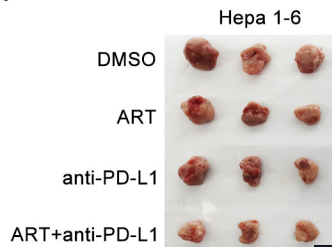

B

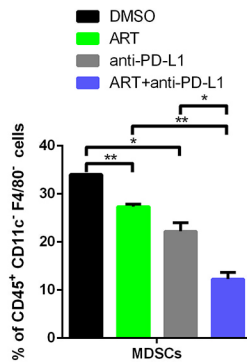

C

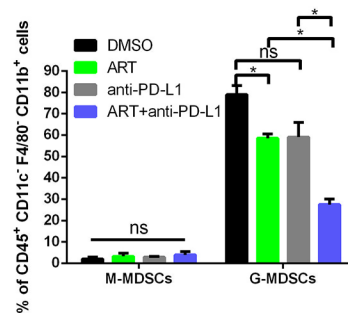

D

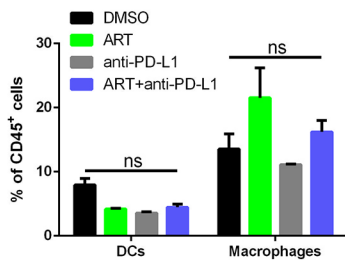

E

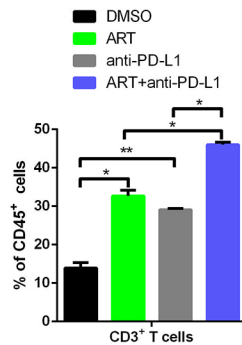

F

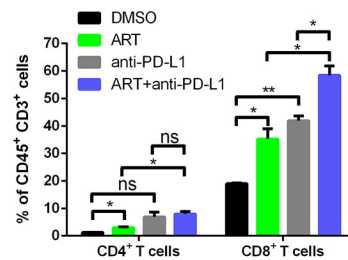

G

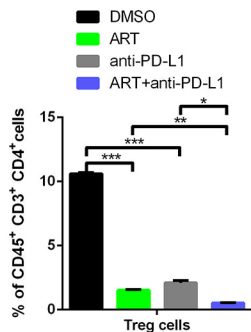

H

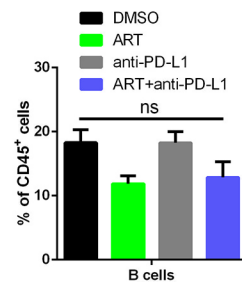

I

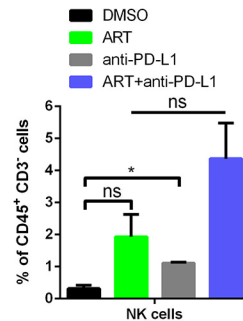

Supplement: Supplementary 5 — Figure S5: targeting MDSCs via ART therapy significantly enhances the efficacy of anti-PD-L1 immunotherapy in tumor-bearing mice. (A–I) C57BL/6 mice injected s.c. on day 0 with Hepa1-6 hepatoma cells and treated with 50 mg/kg ART once every day starting from day 5 while administrated 10 mg/kg anti-PD-L1 antibodies every three days starting from day 5 of Hepa 1-6 tumor model. (A) The image of tumor tissues excised from Hepa 1-6 tumor-bearing mice on day 21. Scale bars, 10 mm. (B–I) Tumor tissues were isolated in the similar tumor volume ~1000 mm3: DMSO on day 12, ART or anti-PD-L1 on day 15, and ART+anti-PD-L1 on day 21. The proportions of immune cells in tumor tissues: MDSCs, M-MDSCs, G-MDSCs, DCs, macrophages, CD3+ T cells, CD4+ T cells, CD8+ T cells, Treg cells, B cells, and NK cells were detected by flow cytometry. Data are means ± SEM and are from a representative experiment of three (B–I). Unpaired Student's t test for (B)–(I). ∗P < 0.05, ∗∗P < 0.01, and ∗∗∗P < 0.001. ns: not significant. [file 2253436.f5.pdf]
