# Supplementary material for: Targeting Inhibition of Accumulation and Function of Myeloid-Derived Suppressor Cells by Artemisinin via PI3K/AKT, mTOR, and MAPK Pathways Enhances Anti-PD-L1 Immunotherapy in Melanoma and Liver Tumors
Source: J Immunol Res. 2022 Jun 22;2022:2253436. doi: 10.1155/2022/2253436 (PMC9247850; doi:10.1155/2022/2253436)
Supplement: Supplementary 6 — Table S1: list of qPCR primer sequences. [file 2253436.f6.pdf]

Family	Gene name(Foverd-F and Reverse-R)	Species	Primer sequence(5'-3')
**MDSC function factors**	*Arg1-F*	mouse	ATTATCGGAGCGCCTTTCTC
	Arg1-R	mouse	ACAGACCGTGGGTTCTTCAC
	Inos-F	mouse	CACCTTGGAAGAGGAGCAAC
	Inos-R	mouse	AAGGCCAAACACAGCATACC
	$P47^{phox}$-F	mouse	CCACACCTGCTGGACTTCTT
	$P47^{phox}$-R	mouse	GCCACGGTCATCTCTGTTC
	$Gp91^{phox}$-F	mouse	AACTGGGCTGTGAATGAAGG
	$Gp91^{phox}$-R	mouse	CAGTGCTGACCCAAGGAGTT
	$P22^{phox}$ -F	mouse	GTGGCTACTGCTGGACGTTT
	$P22^{phox}$ -R	mouse	TGGACCCCTTTTTCCTCTTT
	$P67^{phox}$-F	mouse	AAACTCAGACGCCAGTAAGCA
	$P67^{phox}$-R	mouse	CCAGCCATTCTTCATTCACA
	$P40^{phox}$-F	mouse	GACACAGGCAAAACCATCAA
	$P40^{phox}$-R	mouse	ACAGCAGCCTAACCAAGTCC
	Rac1-F	mouse	GCTGACTCCCATCACCTACC
	Rac1-R	mouse	TCGGATAGCTTCGTCAAACA
	S100a8-F	mouse	GGAAATCACCATGCCCTCT
	S100a8-R	mouse	TTTATCACCATCGCAAGGAAC
	S100a9-F	mouse	AATGGTGGAAGCACAGTTGG
	S100a9-R	mouse	GCTGATTGTCCTGGTTTGTG
	Il6-F	mouse	TAGTCCTTCCTACCCCAATTTCC
	Il6-R	mouse	TTGGTCCTTAGCCACTCCTTC
	Il4ra-F	mouse	CCTCACACTCCACACCAATG
	Il4ra-R	mouse	AGCCTGGGTTCCTTGTAGGT
	Il10-F	mouse	GAAGACCCTCAGGATGCGG
	Il10-R	mouse	ACCTGCTCCACTGCCTTGCT
	Tnfα-F	mouse	CATCTTGTCAAAATTCGAGTGACAA
	Tnfα-R	mouse	TGGGAGTAGACAAGGTACAACCC
	Tgfb1-F	mouse	CGCTGCTACTGCAAGTCAGA
	Tgfb1-R	mouse	GGTAGCGATCGAGTGTCCA
	Il10r2-F	mouse	TTTGTCGTGCTGTGGCTCAT
	Il10r2-R	mouse	AGGGAAGGAGAACAGCAGAA
	Il10r1-F	mouse	CCCATTCCTCGTCACGATCTC
	Il10r1-R	mouse	TCAGACTGGTTTGGGATAGGTTT
	Il12p40-F	mouse	AGGTCACACTGGACCAAAGG
	Il12p40-R	mouse	AGGGTACTCCCAGCTGACCT
	Il6ra-F	mouse	CCTGTGTGGGGTTCCAGAGGAT
	Il6ra-R	mouse	CTGCCAGTATTCTCAGCAGCTG
	Il1a-F	mouse	TCTCAGATTCACAACTGTTCGTG
	Il1a-R	mouse	AGAAAATGAGGTCGGTCTCACTA
